# Prognostic factors in non-surgically treated sciatica: A systematic review

**DOI:** 10.1186/1471-2474-12-208

**Published:** 2011-09-25

**Authors:** Julie Ashworth, Kika Konstantinou, Kate M Dunn

**Affiliations:** 1 Arthritis Research UK Primary Care Centre, Primary Care Sciences, Keele University, Staffordshire, UK

## Abstract

**Background:**

When present sciatica is considered an obstacle to recovery in low back pain patients, yet evidence is limited regarding prognostic factors for persistent disability in this patient group. The aim of this study is to describe and summarise the evidence regarding prognostic factors for sciatica in non-surgically treated cohorts. Understanding the prognostic factors in sciatica and their relative importance may allow the identification of patients with particular risk factors who might benefit from early or specific types of treatment in order to optimise outcome.

**Methods:**

A systematic literature search was conducted using Medline, EMBASE and CINAHL electronic databases. Prospective cohort studies describing subjects with sciatica and measuring pain, disability or recovery outcomes were included. Studies of cohorts comprised entirely of surgically treated patients were excluded and mixed surgically and conservatively treated cohorts were included only if the results were analysed separately by treatment group or if the analysis was adjusted for treatment.

**Results:**

Seven adequate or high quality eligible studies were identified. There were conflicting but mainly negative results regarding the influence of baseline pain severity, neurological deficit, nerve root tension signs, duration of symptoms and radiological findings on outcome. A number of factors including age, gender, smoking, previous history of sciatica and heaviness of work do not appear to influence outcome. In contrast to studies of low back pain and purely surgically treated sciatica cohorts, psychological factors were rarely investigated.

**Conclusions:**

At present, the heterogeneity of the available studies makes it difficult to draw firm conclusions about sciatica prognosis, and highlights the need for further research for this group of patients. Large scale prospective studies of high methodological quality, using a well-defined, consistent definition of sciatica and investigating psychosocial factors alongside clinical and radiological findings are recommended to identify prognostic factors in this population.

## Background

Sciatica is one of the commonest variations of low back pain (LBP) [1] and is considered an obstacle to recovery in LBP patients [2]. In comparison to patients with LBP alone, patients who complain of back and leg pain tend to suffer more severe pain and disability and take longer to recover [3].

There is an extensive body of literature investigating the prognostic factors for LBP. Various socio-demographic, clinical, occupational and psychosocial factors have been identified [2,4-7], although it is acknowledged that individual risk factors explain only a modest part of the variance and combinations of risk factors provide a stronger indication of prognosis [8,9]. In contrast, there are no published reviews of prognosis in non-surgically treated sciatica.

Understanding the prognostic factors in sciatica and their relative importance may allow the identification of patients with particular risk factors who might benefit from early or specific types of treatment in order to optimise outcome. It may also permit the development of conservative treatments directed at those modifiable factors with the greatest influence on outcome.

The term sciatica rather than lumbar radiculopathy is used in this review because of its widespread use in the literature [10]. The purpose of this paper is to systematically review, describe and synthesize the literature investigating the prognostic factors for sciatica.

## Methods

### Search Strategy

Electronic database searches of MEDLINE (1950 - December 2010), EMBASE (1980 - December 2010) and CINAHL (1981 - December 2010) were performed using the keywords: sciatica, lumbar radiculopathy, lumbar radicular pain, lumbosacral radicular syndrome, ischias, ischialgia, lumbar nerve root pain, prognos* (truncated), predict* (truncated), outcome, risk factor, recovery, natural history, cohort study, longitudinal study, prospective study, prognostic study. Hand-searches of reference lists of identified articles and relevant review articles were also conducted.

### Inclusion criteria

Articles were considered eligible for the review if they met the inclusion criteria detailed in Table 1.

**Table 1:** Inclusion criteria

1.	Observational cohort study
2.	Adult study population aged 18 years or over

3.	Study population with symptoms and or signs indicative of 'sciatica' based on individual study criteria, with the broadest accepted definition being "pain down the leg which spreads below the knee"

4.	Outcome measures include one or more of pain, function, disability, recovery or psychosocial measures.

5.	Minimum follow-up period of 3 months

6.	Publication in English

### Exclusion criteria

Studies evaluating a single prognostic factor in isolation were excluded as sciatica prognosis is likely to be multifactorial and therefore best investigated using multivariable approaches [11]. Studies of cohorts comprised entirely of surgically treated patients were also excluded because prognosis post surgery may well be influenced by different factors compared to prognosis following conservative treatment. Mixed surgically and conservatively treated cohorts were included only if the results were analysed separately by treatment group or if the analysis adjusted for treatment.

### Methodological quality assessment

Methodological quality was assessed using a 17-item checklist. Table 2 shows the checklist scoring for each study.

**Table 2:** Methodological Quality Scoring^1 ^for all studies

		Study							
	**Checklist item**	**1. **[12]	**2.**[13]	**3.**[14]	**4.**[15]	**5.**[16]	**6.**[17]	**7.**[19]	**8. **[18]

1	Is there a rationale for the study?	Yes	Yes	Yes	Yes	Yes	Yes	Yes	Yes

2	Is a clear study objective/goal defined?	Yes	Yes	Yes	Yes	Yes	Yes	Yes	Yes

3	Are key elements of study design described (e.g. how were participants identified/recruited)	Yes	Yes	Yes	Yes	Yes	Yes	Yes	Yes

4	Are the setting and selection criteria for the study population described?	Yes	Yes	Yes	Yes	Yes	Yes	Yes	Yes

5	Is the follow-up period appropriate?	Yes	Yes	Yes	Yes	Yes	Yes	Yes	Yes

6	Are there any strategies to avoid loss to follow-up, or address missing data?	No	No	No	No	Yes	No	No	No

7	Is the sample size justified?	No	No	No	No	No	No	No	No

8	Is information presented about the measurement instruments used to measure the prognostic variable(s) and does this enable replication (through the use of standardised or valid measures)?	Yes	Yes	Yes	Yes	Yes	Yes	Yes	Yes

9	Is the outcome selected and assessed appropriately?	Yes	Yes	No	No	Yes	No	Yes	Yes

10	Are the study sample described (demographic/clinical characteristics)?	Yes	Yes	Yes	Yes	Yes	No	Yes	Yes

11	Is the final sample representative of the study's target population?	Yes	Yes	Yes	Yes	Yes	Yes	Yes	Yes

12	Is loss to follow-up ≤ 20%? (If not, are there any significant differences between responders and non-responders to follow-up on baseline variables? If yes, have the implications been considered?)	Yes	Yes	No 28%	Yes	Yes	Yes	Yes	Yes

13	Are the main results reported (including prevalence of prognostic indicator(s) & outcome, strength of association, and statistical significance)?	Yes	Not fully	Not fully	Not fully	Yes	Not fully	Yes	Yes

14	Is the statistical analysis appropriate and described?	Yes	No	Yes	Yes	Yes	No	Yes	Yes

15	Were potential confounders and effect modifiers identified and accounted for (e.g. multivariate analysis)?	Yes	No	Yes	Yes	Yes	No	Yes	Yes

16	Do the findings support the authors' interpretations?	Yes	Yes	Yes	Yes	Yes	Yes	Yes	Yes

17	Do the authors discuss study limitations (e.g. biases/generalisability)?	Yes	Yes	Yes	No	Yes	No	Yes	No

	**Total Score**	**15**	**12**	**12**	**12**	**16**	**8**	**15**	**14**

**Scoring**: Total number of yes answers gives overall score

0-10 = poor quality 11-14 = adequate quality 15-17 = high quality

^1 ^Based on a draft developed by a consensus group who met at the International Forum IX for Primary Care Research on Low Back Pain, in October 2007.

### Review Process

The titles and abstracts from the search were examined independently by two authors (JA, KK). The full papers of potentially eligible articles were examined independently by all three authors. Disagreements were resolved by consensus.

### Data extraction and analysis

Data regarding outcome was extracted according to the criteria used to define outcome in the individual paper. A prognostic factor was considered to be statistically significant if the reported p value was < 0.05 or the 95% confidence interval around an odds ratio (OR) did not include 1.0. Prognostic factors drawn from multivariate rather than univariate analyses are presented for studies where multivariate analysis was carried out.

## Results

**Figure 1:**
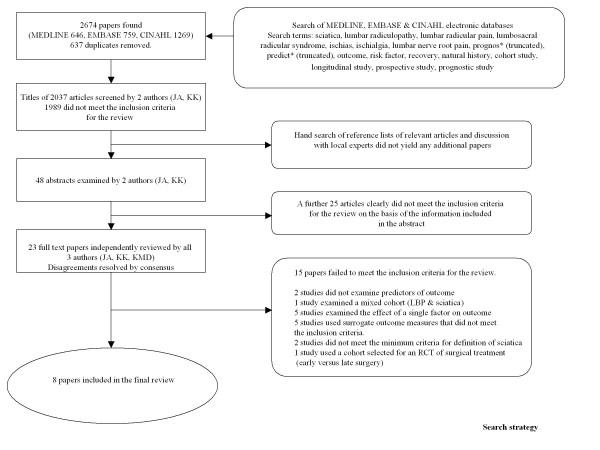
**Search Strategy**.

The search yielded 2674 citations. Eight met all the criteria for inclusion. The process for selecting the eligible studies is shown in Figure 1. The characteristics of the included studies are summarised in Table 3 and the significant prognostic factors from individual studies are presented in Table 4.

**Table 3:** Individual Study Characteristics

ID	Author	Population studied	Subjects	Sciatica definition	Treatment	Follow-up (months)	Study Quality	Predictors studied	Outcomes measured
1	Balague et al (1999) [12]	Consecutive hospital admissions with severe acute sciatica	82 66% male mean age 43 yrs 73 at follow-up	Unilateral leg pain +/- LBP **and** positive neurological signs **and/or** radiological evidence of spinal nerve root compression	Conservative "intensive pain management"	12	High	Age, gender, duration of symptoms, smoking, previous sciatica, EMG, BMI, QOL, disability, pain, imaging results (MRI, CT), neurological signs, antibody test	"Recovery" (composite score including pain, disability & muscle strength) Recovery defined as: ODI Score ≤ 20 VAS pain ≤ 15 Normal muscle strength test (score 5)

2	Beauvais et al (2003) [13]	Consecutive patients attending rheumatology departments with symptoms of sciatica or femoral neuralgia of < 1 month duration and disc herniation on CT	75 58% male mean age 41 yrs 60 at follow-up	Symptoms & examination consistent with sciatic or femoral neuralgia **and** CT evidence of intervertebral disk herniation	Conservative Bed rest, analgesics, lumbar brace +/- epidural steroid injection	3	Adequate	Age, gender, distribution of pain, duration of pain, previous sciatica, presence of severe pain requiring inpatient treatment, CT findings	"Recovery" Complete = return to usual work/activities, little or no analgesia Partial = residual pain, frequent analgesic use, complete or partial return to work, limited athletic activities Failure = persistent pain, continuous analgesic use, unable to return to work
3	Carragee & Kim (1997) [14]	Consecutive patients referred to hospital for MRI scan with symptoms suggestive of sciatica and available for 2 year follow-up	188 58% male mean age 42.5 yrs 135 at follow-up	Lower extremity radicular pain (greater than back pain) **and** Positive SLR test or motor weakness **and** abnormal MRI scan	Usual care Conservative 64% and surgical 36%	24	Adequate	Disc morphology on MRI, age, gender, height, weight, duration, affected side, previous spinal surgery, occupation (heaviness of work), SLR, motor weakness, co-morbidity, smoking, alcohol, workers compensation, litigation, mode of treatment.	Composite measure of overall outcome comprising sum of scores on 0-10 scale for self-reported pain, medication use, activity restriction and satisfaction, total divided by 4 to give outcome score > 6 = good ≤6 = poor
4	Hasenbring et al (1994) [15]	Consecutive patients admitted to hospital with acute radicular pain and radiologically diagnosed disc prolapse	111 60% male mean age 41.7 yrs 90 at follow-up	Acute radicular pain **and** radiologically diagnosed lumbar disc prolapse or protrusion	Usual care Surgical 66% Conservative 34%	6	Adequate	Depression (BDI), "daily hassles in fifteen areas of daily living including work, home, relationships and financial" (KISS) "emotional, cognitive & coping reactions to pain" (KSI), health locus of control", duration of symptoms, nature of onset, previous surgery, disc displacement on imaging, paresis, scoliosis, treatment (surgical/conservative), obesity, age, social status, occupation (posture, heaviness of work), duration of inability to work	Pain Intensity Self report 8 point scale
5	Jensen et al 2007 [16]	Consecutive patients referred to a specialist outpatient back pain centre with symptoms suggestive of sciatica and enrolled in an RCT of active conservative treatment	187 55.5% male mean age 45 yrs 154 at follow-up	Radicular symptoms with a dermatomal distribution	Conservative Education, reassurance, analgesia, +/- exercise programme +/- manual physiotherapy If surgery required patients excluded from follow-up analysis	14	High	MRI findings (disc contour, height, signal & herniation); nerve root compromise; spinal stenosis (central, lateral, foraminal). Age Gender Treatment	"Recovery" (composite score including pain on 11 point VRS & disability on RMDQ) Recovery defined as: Pain score < 1 & RMDQ ≤ 3
6	Komori et al 2002 [17]	Consecutive patients presenting to hospital with unilateral leg pain and with radiologically confirmed herniated disc	131 no demographic data presented 90 at follow-up	Unilateral leg pain **and** MRI evidence of herniated nucleus pulposus	Usual care Conservative - rest, medication, traction. If surgery required patients excluded from follow-up analysis	12	Poor	Age, gender, occupation (heaviness of work), previous LBP or sciatica, Duration of symptoms Leg symptoms ( pain, SLR, FST, motor paresis & sensory disturbance) Level & type of herniation/disc degeneration on MRI scan	Outcome defined according to residual self-reported symptoms and disability on 3 point scale (poor, fair, good)
7	Miranda et al (2002) [19]	Employees of Finnish forestry industry receiving annual questionnaire about musculoskeletal pain	3312 74% male mean age 45.3 yrs 2984 at follow-up	Self-reported low back pain with leg pain radiating below the knee	None	12	High	Age, gender, weight, height, smoking, driving, mental stress Occupational activities (twisting, bending, kneeling or squatting, working with arms raised, lifting), heaviness of work, 'overload' at work, risk of accident at work, Physical exercise and sporting activity in general & specific sports	Outcome defined as persistence of pain based on self report of sciatic pain Persistence = sciatica pain on >30 days/year in 2 consecutive years (1994 & 1995) on modified NMQ)
8	Vroomen et al (2002) [18]	Consecutive patients presenting to GP with 1^st ^episode of sciatica and pain sufficient to justify further therapy. Study performed concurrently with RCT of bed rest	183 56% male mean age 46 yrs 169 at follow-up	Leg pain in dermatomal distribution **and** ≥ 2 of the following: • Increased pain on coughing & sneezing • Sensory loss • Muscle weakness • Reflex loss • Positive nerve root irritation signs	Usual care Surgery if indicated (15%) A second analysis excluding patients who had surgical treatment (n = 156) was performed	3	Adequate	Age, gender, education, living alone, employment, previous sciatica, previous LBP, family history, co-morbidity, smoking, sporting activity, BMI, Duration of symptoms, revised Oswestry score, Roland disability score, MPQ score Leg pain > back pain Pain-related symptoms and examination findings (SLR, FST, paresis, sensory loss, finger to floor distance)	Poor outcome defined as absence of any improvement at 3 months based on self-reported change in symptoms

BDI Beck Depression Inventory

BMI Body Mass Index

CT Computed Tomography

EMG Electromyogram

FST Femoral Stretch test

KISS Kiel Inventory of Subjective Situations

KSI Kiel Pain Inventory

LBP Low back pain

MPQ McGill Pain Questionnaire

MRI Magnetic Resonance Imaging

NMQ Nordic Questionnaire

ODI Oswestry Disability Index

QOL Quality of life

RMDQ Roland Morris Disability Questionnaire

SLR Straight leg raise test

VAS Visual Analogue Score

VRS Verbal Rating Scale

**Table 4:** Significant Prognostic Factors identified in all included studies

ID	First Author	Statistical analysis	Outcomes measured	**Statistically significant**^**a **^**predictors of poor outcome**	Strength of association	**Statistically significant**^**a **^**predictors of good outcome**	Strength of association	Comments
^1^	Balague [12]	Multivariate analysis (stepwise logistic regression)	"Recovery" (composite score including pain, disability & muscle strength) Recovery defined as: ODI Score ≤ 20 VAS pain ≤ 15 Normal muscle strength test (score 5)	Positive neurological examination (Neurotot)	OR 4.3 (95%CI; 1.37, 13.28)			It is unclear whether the odds ratio given is crude or adjusted.

^2^	Beauvais [13]	Recovery and failure groups compared using Fishers test, Chi squared test or Wilcoxon test	"Recovery" Complete = return to usual work/activities, little or no analgesia Partial = residual pain, frequent analgesic use, complete or partial return to work, limited athletic activities Failure = persistent pain, continuous analgesic use, unable to return to work	Hospital admission because of severity of sciatic pain	Not reported			

^3^	Carragee [14]	Multivariate analysis (multiple logistic regression)	Composite measure of overall outcome comprising sum of scores on 0-10 scale for self-reported pain, medication use, activity restriction and satisfaction, total divided by 4 to give outcome score > 6 = good ≤6 = poor	Larger ratio of disc to remaining canal (in conservatively treated patients)	R = 0.50	Shorter duration of symptoms Absence of litigation Younger age	Not reported	Data from surgically and non-surgically treated patients analysed separately. Only data from conservatively treated patients presented

^4^	Hasenbring [15]	Multivariate regression analysis	Pain Intensity Self report 8 point scale	Lesser degree of disc displacement Scoliosis High score for non-verbal pain behaviour Low score for direct search for social support Tendency to ignore pain experience Poor ability to imagine coping with the pain Low social status	β = -0.32 β = 0.15 β = 0.31 β = -0.35 β = 0.29 β = -0.20 β = -0.17			Pain intensity was the only outcome studied. 73 (65.8%) underwent surgical treatment but the analysis adjusted for treatment which was not found to be a significant predictor in this study.

^5^	Jensen [16]	Multivariate analysis adjusted for age, sex and treatment	"Recovery" (composite score including pain on 11 point VRS & disability on RMDQ) Recovery defined as: Pain score < 1 & RMDQ ≤ 3			Broad based disc protrusion Disc extrusion Male gender Absence of canal stenosis (males only)	OR 13.6 (95% CI; 1.9, 95.4) OR 10.6 (95% CI; 1.9, 58.7) OR 2.6 (95% CI; 1.3, 5.0) OR 4.2 (95% CI; 1.2, 14.7)	

^6^	Komori [17]	Non-parametric methods (not further specified)	Outcome defined according to residual self-reported symptoms and disability on 3 point scale (poor, fair, good)	Smaller herniated disc Greater symptom severity at initial assessment	Not reported			The findings of this study should be interpreted with caution due to poor methodological quality

^7^	Miranda [19]	Multivariate logistic regression	Outcome defined as persistence of pain based on self report of sciatic pain Persistence = sciatica pain on >30 days/year in 2 consecutive years (1994 & 1995) on modified NMQ)	Poor job satisfaction Ex-smoker Jogging	OR 2.8 (95% CI; 1.2,6.7) OR 2.3 (95% CI; 1.3,4.3) OR 3.9 ( 95% CI;1.4,10.7)			Diagnosis of sciatica based on self-reported symptoms only

^8^	Vroomen [18]	Multivariate logistic regression	Poor outcome defined as absence of any improvement at 3 months based on self-reported change in symptoms	Duration of pain > 30 days Positive SLR	OR 10 (95%CI;2.5,33.3)* OR 2.5 (95%CI;1.25,20)* * see footnote			Patients undergoing eventual surgery excluded from this analysis. Follow up period only 3 months.

* We have recalculated the odds ratios for poor outcome from the original report of the analysis of patients treated conservatively throughout p < 0.05

The majority of studies were in a secondary care or hospital setting [12-17], one was in primary care [18] and one looked at workers in a community setting [19]. Sample size at follow-up varied from 60 to 2984 and follow-up was for 12 months or more in 5 studies [12,14,16,17,19], 6 months in 1 study [15] and 3 months in 2 studies [13,18]. In terms of methodological quality, 3 studies were rated "high quality" [12,16,19], 4 "adequate quality" [13-15,18] and 1 'low quality' [17] (Table 2). The findings of the low quality study [17] are presented in Table 4 but are excluded from the analysis presented in Table 5 and from the discussion below.

**Table 5:** Prognostic factors reported in 3 or more studies and their association with poor outcome

Prognostic factor studied	Positive association with poor outcome	No association
**Socio-demographic/individual**		

Older age	0	6 [12,13,15,16,18,19]

Gender	0	5 [12-14,18,19]

Previous sciatica	0	3 [12,13,18]

Smoking	0	4 [12,14,18,19]

Higher BMI/obesity (15% overweight)	0	3 [12,15,18]

**Clinical (symptoms & signs)**		

Longer duration of symptoms	1 [18]	3 [12,13,15]

Baseline pain/symptom severity	1 [13*]	2 [12,18]

Neurological deficit	1 [12]	3 [14,15,18]

Nerve root tension signs	1 [18]	2 [12,14]

**Clinical (radiological findings)**		

Level of disc herniation	0	5 [12-16]

Smaller disc prolapse	1 [15]	3 [12-14]

**Occupational**		

Heaviness of work	0	3 [14,15,19]

* Beauvais et al [13] reported that pain/symptom severity sufficient to require inpatient treatment was associated with poor outcome.

NB Komori et al [17] is excluded from this table due to poor methodological quality

There was variation in the definition of sciatica; however, all but one study clinically defined symptoms in ways that are commonly described in the medical literature. One study used self-reported symptoms of 'LBP radiating below knee', a definition often used in epidemiological studies as a proxy for sciatica.

Six out of eight studies reported prognostic factors for poor outcome, one reported prognostic factors for good outcome (recovery) and one study reported prognostic factors for good and poor outcome.

A large number of potential prognostic factors were studied with considerable variation between studies. Overall, 76 individual potential prognostic factors were considered but 30 relate to specific occupational activities or sports from one study [19] and a further 29 are only considered in a single study. The large number of prognostic factors examined in only 1 or 2 studies makes analysis of the results difficult; therefore an overview of the prognostic factors for poor outcome considered in at least 3 studies is presented in Table 5.

### Individual/socio- demographic prognostic factors

Age was considered but not found significant in 6 studies reporting on prognostic factors for poor outcome. In one study considering prognostic factors for good outcome [14], younger age was found to be significant but no strength of association was reported. Gender was considered in 5 [12-14,19,18] out of 6 studies reporting on prognostic factors for poor outcome but none found it significant. One study [16] reporting prognostic factors for good outcome found male gender to be significant (OR 2.6; 1.3, 5.0). Current smoking was not found to be a significant prognostic factor in 4 out of 4 studies [12,14,18,19]. Miranda et al [19] reported worse outcome (persistence of sciatica) in ex-smokers (OR 2.8;1.2,6.7). A previous history of sciatica was not found to be a significant prognostic factor in 4 out of 4 studies [12,13,18,19]. Previous spinal surgery was not found to be a significant prognostic factor in 2 out of 2 studies [14,15].

Obesity was considered in one study [15] and Body Mass Index (BMI) in two others [12,18] but none found either to be a significant prognostic factor. Two further studies [14,19] considered body weight and height but neither was significant. Low social status was identified as a prognostic factor for poor outcome in the only study considering this [15]. Absence of litigation was found to be a prognostic factor for good outcome in the only study considering this [14]. Moderate or active jogging was reported as a prognostic factor for the persistence of sciatica (OR 3.9; 1.4, 10.7) in the only study considering it [19].

Individual prognostic factors considered in a single study were alcohol consumption [14], workers compensation [14], education [18], living alone [18], physical activity [19], various sports [19], driving [19] and family history of sciatica [18] but none was found to be significant.

### Clinical prognostic factors

Three studies investigated baseline pain severity [12,13,18]. One adequate quality study, using hospital admission for treatment as a surrogate measure of pain severity [13], found a significant association with poor outcome but one high quality and one adequate quality study [12,18] did not. Only one [12] of four studies which investigated neurological deficit identified this as a significant prognostic factor of poor outcome. Only one [18] of three studies considering nerve root tension signs reported a positive straight leg raise (SLR) to be associated with poor outcome (OR 2.5 CI;1.25, 20).

One [18] out of four studies considering duration of symptoms found longer duration to be associated with poor outcome. A further study [14] reported shorter duration of symptoms as a significant prognostic factor for good outcome but no strength of association was reported.

Five studies examined the association between radiological findings and outcome. None found the level of disc herniation to be significant. Four of these studies [12-15] reported on prognostic factors for poor outcome. One study [15] found smaller disc herniation to be significant, whilst, another study [14] found that a larger ratio of disc to remaining canal was associated with poor outcome (R = 0.50). The remaining two studies found no association [12,13]. One study [16] found that broad based disc protrusion and disc extrusion were significant prognostic factors for good outcome (OR 13.6;1.9, 95.4 and OR 10.6; 1.9, 58.7, respectively). The presence of scoliosis was associated with poor outcome (beta 0.15, p < 0.011) in the only study to consider this [15]. The absence of spinal stenosis was found to be a prognostic factor for good outcome in males only (OR 4.2; 1.2, 14.7) in one study [16] but was not found to be significant in two further studies [13,14] considering spinal stenosis as a potential prognostic factor for poor outcome.

The remaining clinical prognostic factors were considered only in a single study and none of these (distribution of pain [13], affected side [14], greater leg than back pain [18], ability to bend forwards [18], EMG and antibody test [12] was found to be significant.

### Occupational factors

Three studies [14,15,19] considered the heaviness or strenuousness of work but none found this to be a significant prognostic factor. One study [19] also considered various occupational activities (bending, lifting, twisting, squatting) but none was found to be significant. Poor job satisfaction was associated with poor outcome (OR 2.8; 1.2, 6.7) in the only study which investigated this [19]. The only study [18] to investigate employment status did not find it to be a significant prognostic factor.

### Psychological prognostic factors

Two studies looked at psychological prognostic factors. One study [15] considered a number of psychological factors including depression, 'daily hassles' and cognitive and emotional coping strategies for pain. They found a high score for non-verbal pain behaviour, a low score for direct search for social support, a tendency to ignore pain experience and a poor ability to imagine coping with the pain were all significant prognostic factors for poor outcome. Collectively, the psychological variables measured in this study explained 37% of the outcome variance. Another study [19] examined self-reported 'mental stress' on a 4 point scale and found a high score for mental stress to be a significant prognostic factor for persistence of sciatica in the univariate but not the multivariate analysis.

## Discussion

This is the first systematic review to look at factors affecting sciatica outcome outside purely surgically treated populations. The review found seven eligible studies of adequate or high methodological quality and one of poor quality; their heterogeneity precluded the statistical pooling of results.

This review found conflicting results regarding the association of pain severity with outcome. Duration of symptoms and neurological deficit were found to be significant in only one out of four studies. Conflicting results were found in terms of the influence of the size and type of disc prolapse on outcome. In surgical sciatica cohorts, pain severity, duration of symptoms and neurological deficit have all previously been identified as prognostic factors for poor outcome [20].

The results also suggest a number of factors which do not appear to affect outcome; including age, gender, smoking and heaviness of occupation. In the literature on prognosis for surgically treated sciatica, age and smoking have similarly been reported not to affect outcome, and the evidence for gender and physical work in the surgical population is conflicting [20]. Female gender has also been reported as a poor prognostic factor in some LBP studies although overall the evidence is conflicting [2].

The evidence regarding the prognostic role of psychological factors in conservatively treated sciatica is limited to two studies [15,19] with only one study, which reported pain intensity as the sole outcome, [15] finding them significant. This contrasts with studies of LBP [2,4,6] and also surgically treated sciatica [20] where psychosocial factors are frequently examined and are often found to be associated with outcome, mainly in terms of persistent disability.

Overall, the current existing evidence does not allow firm conclusions to be drawn about the prognostic factors in conservatively treated sciatica. A number of limitations of the included studies may contribute to this. It is suggested that for prognostic studies at least 10 outcome events are required for each factor studied [11]. It is possible therefore that most of the included studies had too small a sample size in relation to the number of predictors studied. Furthermore, in excess of 70 predictors were considered across the various studies, but most were considered in only 1 or 2 studies making it difficult to draw firm conclusions even when significant factors were identified. Large studies investigating all potential prognostic factors are needed to overcome these issues.

The heterogeneity of the included studies presents further difficulties in analysing the results. There is variability in the definition of sciatica with one study using self-reported symptoms, and only 4 studies requiring radiological confirmation of disc prolapse. It is possible therefore that not all subjects had leg pain associated with nerve root involvement. The diverse outcome measures used, ranging from self-reported improvement to complex composite measures of recovery may also have contributed to the conflicting results. Furthermore, three studies only presented p values, which offer little help in appreciating the clinical relevance of a prognostic factor and do not allow for comparison of results between studies.

This systematic review has highlighted the fact that in contrast to prognostic factors for persistent disability for LBP patients and surgically treated patients with sciatica, very little is still known about such factors for the majority of sciatica patients that are treated conservatively. New evidence from studies published following this review may, of course, influence these results.

## Conclusion

The data presented here do not suggest any one strong or consistent predictor of persistent disability in mainly conservatively treated sciatica cohorts. However, a number of factors have been identified that do not seem to significantly affect outcome of sciatica. The results of this review suggest that the prognostic factors for conservatively treated sciatica may differ to some extent from those for surgically treated patients and from LBP in general. However, the heterogeneity of the available studies makes it difficult to draw firm conclusions and demonstrates the need for further research. It is recommended that good quality, prospective, large scale prognostic studies in sciatica cohorts are carried out. These should investigate psychosocial and occupational factors alongside clinical and radiological findings using consistent validated outcome measures for pain, function and recovery and report the strength of association of all prognostic factors identified. Furthermore a consistent definition of sciatica is needed in future studies. A possible solution would be for future studies to include only subjects with clinically diagnosed sciatica and radiological confirmation of lumbar disc herniation.

Sciatica is more disabling and costly than LBP alone and the findings of such epidemiological studies may allow us in future to better predict outcome or likely response to treatment of patients with sciatica, based on the presence of certain characteristics. This may permit the targeting of treatments to particular patient subgroups.

## Abbreviations

BDI: Beck Depression Inventory; BMI: Body Mass Index; CT: Computed Tomography; EMG: Electromyogram; FST: Femoral Stretch test; KISS: Kiel Inventory of Subjective Situations; KSI: Kiel Pain Inventory; LBP: Low back pain; MPQ: McGill Pain Questionnaire; MRI: Magnetic Resonance Imaging; NMQ: Nordic Questionnaire; ODI: Oswestry Disability Index; QOL: Quality of Life; RMDQ: Roland Morris Disability Questionnaire; SLR: Straight leg raise test; VAS: Visual Analogue Score; VRS: Verbal Rating Scale.

## Competing interests

The authors declare that they have no competing interests.

## Authors' contributions

JA and KK carried out the literature search and screening of identified titles and abstracts. All three authors (JA, KK, KMD) reviewed the selected articles, contributed to the development and design of the review and to the writing of the manuscript. The final manuscript was read and approved by all three authors.

## Pre-publication history

The pre-publication history for this paper can be accessed here:

http://www.biomedcentral.com/1471-2474/12/208/prepub
